# Greenhouse Gas Emissions, Energy Consumption and Economic Growth: A Panel Cointegration Analysis for 16 Asian Countries

**DOI:** 10.3390/ijerph14111436

**Published:** 2017-11-22

**Authors:** Wen-Cheng Lu

**Affiliations:** Department of Economics and Finance, Ming Chuan University, 5 De Ming Rd., Gui Shan District, Taipeh 333, Taiwan; luwc@mail.mcu.edu.tw; Tel.: +886-3-350-7001

**Keywords:** greenhouse gas emissions, energy consumption, economic growth, Granger causality

## Abstract

This research investigates the co-movement and causality relationships between greenhouse gas emissions, energy consumption and economic growth for 16 Asian countries over the period 1990–2012. The empirical findings suggest that in the long run, bidirectional Granger causality between energy consumption, GDP and greenhouse gas emissions and between GDP, greenhouse gas emissions and energy consumption is established. A non-linear, quadratic relationship is revealed between greenhouse gas emissions, energy consumption and economic growth, consistent with the environmental Kuznets curve for these 16 Asian countries and a subsample of the Asian new industrial economy. Short-run relationships are regionally specific across the Asian continent. From the viewpoint of energy policy in Asia, various governments support low-carbon or renewable energy use and are reducing fossil fuel combustion to sustain economic growth, but in some countries, evidence suggests that energy conservation might only be marginal.

## 1. Introduction

Carbon dioxide emissions from fossil fuel combustion, associated with different economic activities, are an important driver of global climate change. Increasing energy demand stimulates economic growth, but energy use also causes greenhouse gas emissions. In the past decade, increasing attention to global warming and climate change has focused on the relationship between environmental pollutants, energy consumption and economic growth. Many countries are engaged in the mitigation of greenhouse gas emissions. The world’s top energy-consuming countries have widely discussed CO_2_ reduction targets and tabled more sustainable policy interventions aimed at fostering cleaner economic development trajectories (Wang and Wang [1]; Jiang et al. [2]; Wang and Chang [3]; Salahuddin and Gow [4]). To effectively control greenhouse gas emissions and ensure the sustainability of economic development, it is important to better understand the links between greenhouse gas emissions, energy consumption and economic growth.

In the literature, there have been two streams of research exploring the relationship between greenhouse gas emissions, energy consumption and economic growth. The first stream of research focuses on the relationship between environmental pollutants and economic growth and suggests an inverted U-shaped relationship between environmental degradation and real GDP per capita (hereafter, GDP). This relationship is known in the literature as the Environment Kuznets Curve (EKC). Several studies have examined the linkages between economic growth and environmental pollutants. Recent studies focusing on greenhouse gas emissions or CO_2_ emissions more specifically include Hamit-Haggar [5], Li et al. [6], Mercan and Karakaya [7], Chang [8], Akbostanci et al. [9], Dinda and Coondoo [10], Zhang and Cheng [11]. Study conclusions reveal that there is no consistent relationship between the variables to the extent that the evidence in favor of the environmental Kuznets curve represented by an inverted-U function is equivocal at best. Results are highly contingent upon regional- and country-level specificities. The second stream of research investigates the relationship between energy consumption and economic growth. The relationship between energy consumption and economic growth provides insights with respect to the role of energy consumption in economic development. Representative studies in this regard are Soytas and Sari [12], Apergis and Payne [13,14], Ozturk and Acaravci [15], Shahbaz and Lean [16], Omri and Kahouli [17] and Yuan et al. [18]. Study results and causal relationships in the issue of energy consumption and economic growth also vary depending on the choice of dataset, model specification and econometric technique.

**Figure 1:**
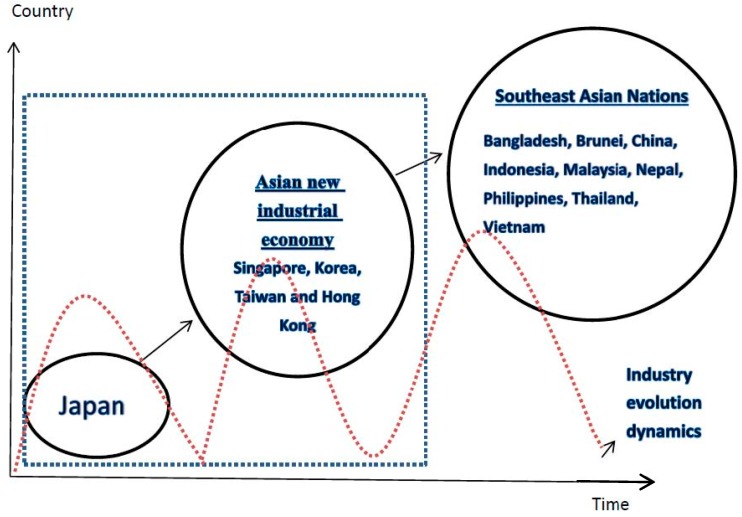
Industry evolution dynamics. Note: As Kyoshi [28] mentioned, we explicitly account for the extent of economic development and divide countries into three groups: first, Japan; then Newly-Industrializing Economies (NIEs) including Korea, Hong Kong, Singapore and Taiwan; second, southeast countries plus China; third, the other regions, mainly Middle Eastern nations, in Asia.

This paper contributes to the literature by extending the analysis of long-run relationships and causal dynamics between greenhouse gas emissions, energy consumption and economic growth to a panel of 16 Asian countries over the period 1990–2012. To the best of our knowledge, there has thus far been no attempt to investigate the relationship between these variables within a trivariate framework across these 16 Asian countries. Previous published studies with regional sampling frames have posited that the relationship between pollutants, energy consumption and economic growth is described in terms of inverted-U functions, in which pollutants initially rise and then decline with respect to income (Apergis, [19]; Christopher et al. [20]; Farhani et al. [21]; Bilgili et al. [22]; Cho et al. [23]; Youssef et al. [24]; Olubenga et al. [25]) Environmental pollutants such as greenhouse gas emissions depend on the extent of economic development. Grubb et al. [26] pointed out that developed economies more generally show divergences between GDP and emissions with no clear linkage between the former and the latter over the last two decades. Thus, we need to delineate our sample into several regions/groups by economic development. The flying geese model (Akamatsu [27]) aims to explain catching-up industrialization processes in latecomer Asian economies (Developing countries adopt suitable labor-intensive or energy-intensive industries from developed countries. Consistent with the flying geese theory, Japan produces first for the home market and then starts to export as soon as industries have matured enough. Initially, the products are simple, crude and cheap, but gradually, the level of quality is elevated. The procedure is repeated over and over again, leading to a rapid process of national development. In the Japanese context, some industries lost their comparative advantage and then moved to less developed Asian countries.) (Kyoshi [28]). According to that framework, Asian countries can be classified into three groups: a lead goose (Japan) to follower geese (Newly-Industrializing Economies (NIEs)) and ASEAN (Association of Southeast Asian Nations) plus China. The dynamic evolution of economic development according to the flying geese theory is illustrated in Figure 1. As mentioned, we explicitly account for the extent of economic development and divide countries into three groups: first, Japan; then, NIEs including Korea, Hong Kong, Singapore and Taiwan; Second, southeast countries plus China. We can investigate the long-run and short-run causal relationships between greenhouse gas emissions, energy use and economic growth by virtue of this three-group country classification vis-à-vis the results and conclusions in the extant literature.

The objective of this paper is to improve upon the extant literature by examining the short-run and the long-run relationships between greenhouse gas emissions, energy consumption and economic growth in 16 Asian countries. We found that a clear long-run cointegration relationship exists between those variables. For the whole sample of 16 Asian countries, the evidence suggests a long-run relationship from energy consumption and GDP to greenhouse gas emissions and from GDP and greenhouse gas emissions to energy consumption. A 1% increase in energy consumption stimulates a: 0.82% increase in greenhouse gas emissions for the 16 Asian countries; a 0.180% increase in greenhouse gas emissions for newly-industrialized countries; and a 0.87% increase for Southeast Asian nations. The non-linear EKC hypothesis holds for the whole 16-country sample. Regionally, the EKC hypothesis also holds for newly-industrialized countries. Short-run causal relationships are regionally contingent. For the 16 Asian countries, we revealed three bidirectional short-run causal relationships: between energy consumption and greenhouse gas emissions, between GDP and energy consumption and between GDP and greenhouse gas emissions. It appears that GDP and energy consumption have a substantive impact on greenhouse gas emissions. Energy consumption also plays an important role in economic growth, and these results imply that energy conservation policies could adversely affect the rate of economic growth, causing decline in that growth. To militate against this, clean and renewable energy policy initiatives that facilitate the growth of solar, hydro and wind power are needed. Short-run causality relationships for Southeast Asian nations are similar to those of the whole country sample; those countries face a feedback effect, which implies that a reduction in energy consumption may adversely affect economic growth. We revealed three bidirectional short-run causal relationships: between energy consumption and greenhouse gas emissions, between GDP and energy consumption and between GDP and greenhouse gas emissions. The results for Asian newly-industrialized countries suggest bidirectional causality between GDP growth and greenhouse emissions and unidirectional causality from energy consumption and greenhouse gas emissions. Those facts suggest that governments have to encourage cleaner and renewable energy use to promote economic growth and abolish energy subsidy and energy price distortion. 

The paper is organized as follows. A brief literature review is offered to provide context and to position the research within the existing literature in Section 2. The data used in this study are identified and described, and our econometric methodology is specified in Section 3. The empirical results associated with our inferential methodology are described and discussed in Section 4; and finally, some conclusions are offered in Section 5.

## 2. Literature Review

Numerous empirical studies have been carried out focusing on energy/electricity consumption and economic growth over the past 20 years. The majority of these studies use time series models to test causal and cointegrating relationships between energy consumption and economic growth within a trivariate or bivariate econometric framework. We focus on the results of cross-country research in this literature review section. Such literature is characterized by conflicting results. For instance, Abdoli et al. [29] use a panel cointegration method to test the existence of causality between economic growth and energy consumption in (Organization for Economic Co-operation and Development) OECD countries over the period 1980–2011. The authors conclude that energy consumption unidirectionally Granger-causes GDP growth in the short-run, but bidirectional causality is established in the long-run. Other studies have investigated different regions that include 10 developing Asian countries (Chen et al. [30]), 21 emerging countries (Bayar [31]), three income-based country groups, namely high-, middle, and low-income countries (Apergis and Payne [32]), 19 European countries (Acaravci and Ozturk [33]), the top 38 renewable consumption countries (Bhattacharya et al. [34]) and 17 African countries (Wolde-Rufael [35]).

Another branch of literature focusing on the energy-growth nexus considers interactions between environment quality or environmental pollutants. The famous and widely-examined theory, the inverted U-shaped environmental Kuznets curve, is relevant in this respect. According to Kuznet’s hypothesis, environmental pollutants are expected to decrease when a certain level of income is reached. The EKC relationship suggests that as development and industrialization progress, environmental damage increases due to greater use of natural resources and higher emissions of pollutants. However, as economic growth continues, people make choices to spend their incomes on cleaner technologies and environmental quality. Behind this relationship, affluence (defined as per capita income or per capita production) is an important determinant of environment pollutants.

Relevant empirical studies are summarized in Table 1. To test the EKC hypothesis, most previous studies have utilized GDP and GDP squared as independent variables. These two variables are incorporated to permit the identification of an inverted-U-shaped relationship between the pollution indicator and GDP. Empirical studies published for different countries or regions are as follows: France (Iwata et al. [36]), China (Zhang and Cheng [11]; Li et al. [6]; Duan et al. [37]), the United States of America (Plassmann and Khanna [38]), Malaysia (Lau et al. [39]; Saboori and Sulaiman [40]), Tunisia (Fodha and Zaghdoud [41]; Farhani et al. [21]), Vietnem (Al-mulai et al. [42]), Indonesia (Sugiawan and Managi [43]), Turkey (Shahbza et al. [44]; Yavuz [45]; Lean and Smyth [46]); Spain (Sephton and Mann [47]), 15 European countries (Apergis [19]), 47 African countries (Christopher et al. [20]), 10 Middle Eastern and North African Countries (MENA; Farhani et al. [21]), 17 OECD countries (Bilgili et al. [22]; Cho et al. [23]), 56 countries (Youssef et al. [24]), 30 administrative regions in China (Li and Ma [48]), 21 Canadian industrial sectors (Hamit-Haggar [5]), 8 countries in Asia and Africa (Olubenga et al. [25]), Central America (Apergis and Payne [13]), Latin America and Caribbean countries (Pablo-Romero and Jesús [49]).

From the forgoing studies, a number of gaps in the literature can be identified. Firstly, insufficient attention has been paid to Asian case-studies. Many countries in Asia are growing rapidly, and as such, the relationship between economic growth and environmental deterioration could be highly dynamic. 

## 3. Econometric Methodology

To investigate the long-run relationship between greenhouse gas emissions, energy consumption and real GDP growth, we propose a framework based on the conventional Environment Kuznets Curve (EKC) hypothesis. The long-run relationship between the aforementioned variables can be specified as follows:

(1) $${\mathrm{GHG}}_{\mathrm{it}}=\alpha_{\mathrm{it}}+\beta_{1i}{\mathrm{ENU}}_{\mathrm{it}}+\beta_{2i}{\mathrm{GDP}}_{\mathrm{it}}+\beta_{3i}{\mathrm{GDP}}_{\mathrm{it}}^{2}+\varepsilon_{\mathrm{it}}$$

The subscript i=1,2, …,N denotes countries while t=1,2,…,T denotes time period (year). GHG denotes greenhouse gas emissions per capita. ENU is energy consumption per capita. GDP and ${\mathrm{GDP}}^{2}$ are real GDP per capita and the square of real GDP per capita, respectively. All variables are expressed in their natural logarithmic forms. The parameters $\beta_{1}$, $\beta_{2}$ and $\beta_{3}$ can be interpreted as elasticities of GHG emissions with respect to energy consumption, real GDP and squared real GDP, respectively. According to the EKC hypothesis, $\beta_{1}$ is expected to be positive so that increasing energy consumption results in increasing emissions. The EKC hypothesis also posits that as economic output increases, GHG emissions increase until a certain level of output is attained after which emissions start to decline. Therefore, $\beta_{2}$ is expected to be positive, whereas $\beta_{3}$ should be negative if this inverted-U theorization of the relationship between economic growth and GHG emissions applied in this empirical context. 

### 3.1. Panel Unit Root Tests

In this section, the stationarity properties of the variables are tested. Traditional univariate unit root tests, initiated by Dickey and Fuller, have low power problems, and it becomes difficult to reject the null hypothesis; because of the weakness of these tests, researchers have exploited the panel dimension of available data in certain applications. We conduct Im, Pesaran and Shin (IPS) [51] unit root tests. The basic equation for IPS panel unit root tests is as follows:

(2) $$\Delta y_{I,t}=\alpha_{i}+\rho_{i}I+\sum_{j=1}^{p}\varphi_{\mathrm{ij}}\Delta I_{i,t-j}+I;\;i=1,2,\ldots ,N;t=1,2,\ldots ,T\;$$

where $y_{i,t}$ represents each variable under consideration in our model. $\alpha_{i}$ is the individual fixed effect, and p is selected so that the residuals are uncorrelated over time. The null hypothesis is that $\rho_{i}=0$ for all i versus the alternative hypothesis that $\rho_{i}<0$ for some $i=1,2,\ldots ,N_{1}$ and $\rho_{i}=0$ for $i=N_{1}+1,\;\ldots ,N$. The IPS statistic is based on averaging individual Augmented Dickey-Fuller (ADF) statistics and can be written as $\bar{t}=\frac{1}{N}\sum_{i=1}^{N}t_{I,T}$, where I is the ADF t-statistic for country i based on the sector-specific ADF regression. IPS reveals that under the null hypothesis of non-stationarity, the $\bar{t}$ statistic follows the standard normal distribution asymptotically. The standardized statistic $t_{\mathrm{IPS}}$ is expressed as:

(3) $$t_{\mathrm{IPS}}=\frac{\sqrt{N\left[\bar{t}-E\left(t_{i}\right)\right]}}{\sqrt{\mathrm{Var}\left(t_{i}\right)}}$$

A limitation of the IPS test is that it is cross-section dependent, potentially because of unobserved common factors, externalities, as well as regional and macroeconomic linkages. Thus, alternative panel unit root tests, addressing cross-section dependence, have been reported. A well-known test, considering cross-section dependence, is the cross-sectional augmented panel unit root (hereafter, CIPS (the cross-sectional augmented panel unit root test)) test after Pesaran [52], where the following Cross-sectional Augmented Dickey–Fuller (CADF) regression is considered and the OLS method for the i-th cross-section in the panel is estimated:

(4) $$\Delta y_{i,t}=\alpha_{i}+\rho_{i}y_{i,t}+\delta_{i}{\bar{y}}_{t-1}+\overset{k}{\underset{j=0}{\sum }}\delta_{\mathrm{ij}}\Delta {\bar{y}}_{i,t-j}+\overset{k}{\underset{j=0}{\sum }}\varphi_{\mathrm{ij}}\Delta y_{i,t-j}+\epsilon_{i,t}$$

where ${\bar{y}}_{t\;-\;1}\;=\;\frac{1}{N}\sum_{i\;=\;1}^{N}y_{i,t\;-\;1}$ and $\Delta {\bar{y}}_{t}\;=\;\frac{1}{N}\overset{N}{\underset{i\;=\;1}{\sum }}\Delta y_{i,t}$. Pesaran [52] proposes a cross-sectional augmented version of the IPS test:

(5) $$\mathrm{CIPS}=\frac{1}{N}\overset{N}{\underset{i=1}{\sum }}{\mathrm{CADF}}_{i}$$

where ${\mathrm{CADF}}_{i}$ is the CADF statistic for the i-th cross-sectional unit given by the t-statistic of the estimate of $\rho_{i}$. The results are reported in Table 2. 

### 3.2. Panel Cointegration Estimation

To test for cointegration among the variables, we employed the heterogeneous panel cointegration test proposed by Pedroni [53,54]. The panel cointegration test allows for cross-sectional interdependence with both different individual effects, and the deterministic trend can be defined as follows:

(6) $${\mathrm{GHG}}_{\mathrm{it}}=c_{\mathrm{it}}+\beta_{1i}{\mathrm{ENU}}_{\mathrm{it}}+\beta_{2i}{\mathrm{GDP}}_{\mathrm{it}}+\beta_{3i}{\mathrm{GDP}}_{\mathrm{it}}^{2}+\varepsilon_{\mathrm{it}}\;\varepsilon_{\mathrm{it}}=\rho_{\mathrm{it}}\varepsilon_{\mathrm{it}-1}+u_{\mathrm{it}}$$

where i=1,2,…,N denotes countries, while t=1,2,…,T denotes time period. GHG represents greenhouse gas emissions per capita. EN is energy consumption per capita. GDP and ${\mathrm{GDP}}^{2}$ represent real GDP per capita and the square of real GDP, respectively. The parameters $c_{\mathrm{it}}$ allow for the possibility of country-specific effects. We expect that $\beta_{1}$ is positive, whereas a negative sign is expected for $\beta_{3}$.

Pedroni [53,54] proposed two types of cointegration tests: panel tests and group tests. First, the panel tests include four statistics, namely panel v-statistic, panel rho-statistic, panel pp-statistic and panel ADF-statistic. These four statistics are based on the within-dimension method. Second, the group tests based on the between-dimension method include three statistics, namely, group rho-statistic, group pp-statistic and group ADF-statistic. In total, these seven statistics (panel tests and group tests) are asymptotically distributed as standard normal; detailed descriptions of panel cointegration test statistics can be found in Pedroni [53]. After establishing panel cointegration, we use Fully-Modified OLS (FMOLS) developed by McCoskey and Kao [55] and Phillips and Moon [56] to estimate parameters. FMOLS is widely used to estimate the long-run relationships among nonstationary variables in a balance panel framework because the OLS estimator has an asymptotic bias. This estimator corrects the serial correlation problems and endogeneity of traditional OLS estimators. The Monte Carlo simulation results of Kao and Chiang [57] show that the OLS estimator has a significant bias with a small N and T. Pedroni [58] also proposed new methods for estimating parameters in econometric models. The technical details of estimation procedures may be found in Pedroni [58]. FMOLS also perform well in finite panels with heterogeneous dynamics and are widely applied. Pedroni [58] extends the modified ordinary least squares estimator (FMOLS) to a panel setting. The FMOLS estimator is constructed by:

$$\beta_{\mathrm{FMOLS}}={\left[\overset{N}{\underset{i=1}{\sum }}\overset{T}{\underset{t=1}{\sum }}X_{\mathrm{it}}X_{\mathrm{it}}^{\prime }\right]}^{-1}\left(\overset{N}{\underset{i=1}{\sum }}\overset{T}{\underset{t=1}{\sum }}X_{\mathrm{it}}{\bar{y}}_{\mathrm{it}}^{+}-\gamma_{12}^{+\prime }\right)$$

where the $X_{\mathrm{it}}$ and $y_{\mathrm{it}}$ are variables that are cointegrated for the panel. ${\bar{y}}_{\mathrm{it}}^{+}$ is the modified dependent variable and the corrected serial correlation terms (i.e., ${\bar{y}}_{\mathrm{it}}^{+}=\left(y_{\mathrm{it}}-{\bar{y}}_{\mathrm{it}}^{}\right)-{\hat{w}}_{12}{Ω}_{22}^{-1}\Delta_{22}$, where Ω and Δ are the estimates of long-run corariances; $\gamma_{12}^{+}=r_{12}-{\hat{w}}_{12}{Ω}_{22}^{-1}\Delta_{22}$). Delete one half bracket and make it suitable.

### 3.3. Granger Causality Tests

After examining the existence of cointegration, the direction of causal relationships between the variables needs to be established. Therefore, we test the Granger causality between greenhouse gas emissions, energy consumption and economic growth. According to the Granger representation theorem, the dynamic vector error correction model can be constructed as follows:

(7) $$\Delta {\mathrm{GHG}}_{i,t}=\mu_{1i}+\sum_{j=1}^{p}\Delta {\mathrm{GHG}}_{i,t-j}\beta_{1j}+\sum_{j=1}^{p}\Delta {\mathrm{ENU}}_{i,t-j}\gamma_{1j}+\overset{p}{\underset{j=1}{\sum }}\Delta {\mathrm{GDP}}_{i,t-j}\delta_{1j}+\;\tau_{1}{\mathrm{ECT}}_{1,\mathrm{it}-1}+\varepsilon_{1,\mathrm{it}}$$

(8) $$\Delta {\mathrm{EN}}_{i,t}=\mu_{1i}+\sum_{j=1}^{p}\Delta {\mathrm{GHG}}_{i,t-j}\beta_{2j}+\sum_{j=1}^{p}\Delta {\mathrm{ENU}}_{i,t-j}\gamma_{2j}+\overset{p}{\underset{j=1}{\sum }}\Delta {\mathrm{GDP}}_{i,t-j}\delta_{2j}+\tau_{2}\;{\mathrm{ECT}}_{2,\mathrm{it}-1}+\varepsilon_{2,\mathrm{it}}$$

(9) $$\Delta {\mathrm{GDP}}_{i,t}=\mu_{2i}+\sum_{j=1}^{p}\Delta {\mathrm{GHG}}_{i,t-j}\beta_{3j}+\sum_{j=1}^{p}\Delta {\mathrm{ENU}}_{i,t-j}\gamma_{3j}+\overset{p}{\underset{j=1}{\sum }}\Delta {\mathrm{GDP}}_{i,t-j}\delta_{3j}\;+\tau_{3}{\mathrm{ECT}}_{3,\mathrm{it}-1}+\varepsilon_{1,\mathrm{it}}$$

where Δ denotes first differencing and ${\mathrm{ECT}}_{1,\mathrm{it}-1}$, ${\mathrm{ECT}}_{2,\mathrm{it}-1}$ and ${\mathrm{ECT}}_{3,\mathrm{it}-1}$ are error correction terms. The lag length is chosen optimally using the Akaike information criterion. The error correction model to be estimated by the Pooled Mean-Group method (PMG) was suggested by Pesaran, Shin and Smith [59,60]; here, we use the PMG estimator to estimate Equations (7) and (9) and evaluate two relationships in Granger causality terms. 

The sources of causation can be identified by testing for the significance of the coefficients of the dependent variables in Equations (7)–(9). The null hypothesis of short-run Granger causality can be shown as follows: (1) short-run causality between GDP and greenhouse gas emissions is tested based on $H_{0}:\delta_{1j}=0,\forall j=1,\ldots ,k,\;\mathrm{in}\;\mathrm{Equation}\;\left(7\right)$ and $H_{0}:\beta_{3j}=0,\forall j=1,\ldots ,p,\;\mathrm{in}\;\mathrm{Equation}\;\left(9\right)$; (2) short-run causality between energy consumption and GDP is tested based on $H_{0}:\delta_{2j}=0,\forall j=1,\ldots ,k$ in Equation (8) and $H_{0}:\gamma_{3j}=0,\forall j=1,\ldots ,p,$ in Equation (9); (3) short-run causality between energy consumption and greenhouse gas emissions is tested based on $H_{0}:\gamma_{1j}=0,\forall j=1,\ldots ,k,\;\mathrm{in}\;\mathrm{Equation}\;\left(8\right)$, and $H_{0}:\beta_{2j}=0,\forall j=1,\ldots ,p,\;\mathrm{in}\;\mathrm{Equation}$ (9). Short-run Granger causality can be tested through a pairwise Granger causality test. For long-run causality, parameter estimation based on the PMG estimator, we can test $H_{0}:\tau_{1}=0,\forall j=1,\ldots ,k,$ in Equation (7), $H_{0}:\tau_{2}=0,\forall j=1,\ldots ,k$ in Equation (8) and $H_{0}:\tau_{3}=0,\forall j=1,\ldots ,k,$ in Equation (9). (The number “(3)” is needed and add it please)

### 3.4. Data

This study uses annual time series data for one developed country including Japan; four newly-industrializing and developing Asian countries, which include Singapore, Hong Kong, Korea, Taiwan; Southeast Asian nations including China, Bangladesh, India, Indonesia, Malaysia, the Philippines, Thailand, Vietnam, Brunei, Nepal and Sri Lanka. Annual data for real GDP per capita, greenhouse gas emissions per capita and energy use per capita are obtained from the World Development Indicators, except Hong Kong and Taiwan. Annual data for real GDP per capita, energy use per capita and greenhouse gas emissions per capita in Taiwan are taken from the Directorate-General of Budget, Accounting and Statistics, and Environmental Protection Administration, Executive Yuan, R.O.C. (The websites are http://eng.dgbas.gov.tw/mp.asp?mp=2 and http://web.epa.gov.tw/en/index.aspx, respectively). Annual data for greenhouse gas emissions per capita in Hong Kong are taken from the Environmental Protection Department of the Government of Hong Kong Special Administrative Region (The website is http://www.epd.gov.hk/epd/english/top.html). Output (GDP) is measured using per capita real GDP in constant 2000 US$, while greenhouse gas emissions per capita (GHG) are expressed in million tons carbon dioxide equivalent (MtCO_2_e). Energy use (EN) is expressed in kg of oil equivalent per capita. The period studied is dependent on the availability of data, and thus, the time period we use is 1990–2012. All variables are transformed into natural logarithms before performing the empirical analysis. All data used and analyzed are on a per capita basis herein. Natural logarithmic variables have a mechanistic value in economics because they approximate elasticities, or growth, of the respective differenced variables. Table 2 presents descriptive statistics. 

## 4. Empirical Results

Our empirical estimation has two objectives. The first is to examine how the variables are related in the long-run. The second is to identify causal relationships between variables. To accomplish these objectives, we first carry out panel unit root tests to test whether all variables are integrated of order one in levels before employing panel cointegration techniques. In the next step, panel cointegration tests are utilized and the Fully-Modified OLS (FMOLS) technique is employed.

### 4.1. Panel Unit Root Test Results

To proceed with cointegration and Granger causality methods, we need to test the order of integration of the data series used. When the series are integrated of the same order, we can proceed with cointegration tests. We employ panel unit root tests after Im, Pesaran and Shin [51] to determine if all variables in the dataset are stationary. Table 3 reports the results of panel unit root tests. These tests indicated that the four time series are nonstationary (i.e., I(1)). However, the unit root can be rejected in first differences for the fourth time series at the 5% level of significance. We can reasonably conclude that those four variables appear to be I(1) series in all countries. 

### 4.2. Panel Cointegration Test Results

The results of the panel cointegration test statistics are listed in Table 4. Seven statistics significantly reject the null hypothesis of no cointegration, with the exception of the group rho-statistic for all sample countries. We found the existence of cointegration relationships among greenhouse gas emissions, energy consumption, GDP and the square of GDP. The analyzed variables trend together in the long-run, so we can state that there is a long-run relationship between greenhouse gas emissions, energy consumption, GDP and the square of GDP for all countries in our sample. 

Table 5 presents the results of the FMOLS estimation. The FMOLS estimate of energy consumption with respect to greenhouse gas emissions is 0.82 and statistically significant at the 5% level for the whole sample of 16 countries. This result indicates that an increase in energy consumption tends to promote greenhouse gas emissions. The coefficient of GDP is positive, but statistically insignificant; this shows that in the long-run, an increase in GDP will tend to weakly expand greenhouse gas emissions. Finally, the coefficient of squared GDP is −0.03, but insignificant at the 5% level. The positive sign for GDP and the negative sign for squared GDP supports the notion that as output increases, GHG also increase until a certain level of output is attained, after which emissions start to decline. The EKC hypothesis does not hold for these 16 Asian countries.

With respect to the results for the Asian newly-industrialized economy sample, the panel FMOLS estimators reveal that the relationship GDP and energy consumption is positive and statistically significant. An increase in GDP and energy consumption for the Asian new industrial economy countries will boost greenhouse gas emissions. The coefficient of squared GDP is −0.4 and significant at the 5% level. The positive sign for GDP and negative sign for squared GDP support the EKC hypothesis that greenhouse gas emissions initially increase with GDP and then decrease after GDP reaches a certain level.

For the Southeast Asian economy sample, the panel FMOLS estimators show that a 1% increase in energy consumption increased greenhouse gas emissions by 0.87%, while a 1% increase in GDP increased greenhouse gas emissions by 0.22%. The coefficient of squared GDP is −0.007 and insignificant at the 5% level. The EKC hypothesis therefore does not hold for the Southeast Asian economy countries. For the sample of other countries in Asia, the panel FMOLS estimators show that a 1% increase in energy consumption increased greenhouse gas emissions by 0.39%, while a 1% increase in GDP increased greenhouse gas emissions by 0.36%. The coefficient of squared GDP is −0.009 and insignificant at the 5% level. The EKC hypothesis also holds for these countries.

The results thus testify to regional heterogeneities. In other words, some regions support the EKC hypothesis, while others do not. It is clear that energy use is an important determinant of greenhouse gas emissions. The results, excepting Southeast Asian nations, indicate that the environment degrades as income increases, until the threshold point is reached. These findings are consistent with those reported for a panel of six Central American countries by Apergis and Payne [13], for five ASEAN countries by Lean and Smyth [46], for 8 countries in Asia and Africa by Olubenga et al. [25], for 47 African countries by Christopher et al. [20] and for 17 OECD countries by Bilgiliet et al. [22].

### 4.3. Panel Granger Causality Test Results

Once the long-run dynamics are established among variables, the next step is to reveal the direction of causality in the short-run. The directions of causality are reported in Table 6. For the whole 16-country sample in Panel A of Table 6, we find three bidirectional causal relationships: energy consumption causes greenhouse gas emissions, and GDP causes greenhouse gas emissions in the short-run. It appears that GDP and energy consumption have a great impact on greenhouse gas emissions. The causal relationships imply that renewable energy policies would be needed to foster sustainable economic development. Short-run causality between GDP and energy consumption may imply that energy conservation policy and clean energy consumption might weakly be achieved. From Panel B of Table 6, the Asian new industrial economy presents a unidirectional causal relationship running from energy consumption to greenhouse gas emissions and a bidirectional causal relationship between GDP and greenhouse gas emissions in the short-run. These results imply that the use of more energy affects GDP growth, resulting in increases in greenhouse gas emissions. These countries need to deploy energy conservation strategies or renewable energy plans to mitigate pollutant emissions, which, at the same time, do not impede economic growth.

Panel C of Table 6 shows the existence of three bidirectional relationships between energy consumption and GDP, between energy consumption and greenhouse gas emissions and between economic growth and greenhouse gas emissions. The results reflect that electricity conservation policies may adversely affect the rate of economic growth and, in turn, cause a decline in economic growth and, thus, lower the demand for energy. This suggests that economic development and energy use lead to greenhouse gas emissions and that energy consumption boosts economic growth. Environmental policy in Southeast Asian nations has to develop renewable energy or promote energy efficiency to reduce greenhouse gas emissions and maintain environmental quality and economic growth. The results for these Southeast Asian nations are similar to those of the whole 16-country sample.

With respect to long-run causality, we test the significance of the coefficients related to error correction terms. The error correction terms denote the speed of adjustment. For Panel A (all 16 countries), the coefficient of −0.08 is significant at the 5% level; this suggests that a deviation from the long-run equilibrium level of greenhouse gas emissions in a year is corrected by 8% over the following year. At the moment, the coefficient of −0.21 with respect to energy consumption is significant at the 5% level, implying that energy consumption responds to deviations from the long-run equilibrium. Therefore, a significant error correction confirms the existence of a stable long-run relationship among greenhouse gas emissions, energy consumption and GDP. Similar results can be found in Panel B and Panel C of Table 6. Both greenhouse gas emissions and energy consumption respond to deviations from the long-run equilibrium. In regard to the results of the whole sample or two subsamples, greenhouse gas emissions and energy consumption exhibit strong long-run causal relationships.

As discussed above, this study find a causal relationship between energy consumption, economic growth and greenhouse gas emissions. The feedback hypothesis is found in the sample of 16 countries (especially in Southeast Asian countries) and a bidirectional causal relationship holds between energy consumption and greenhouse gas emissions. The policy implications in Southeast Asia countries can be categorized into four sectors: industry, transport, building and power. For the industry sector, the government can encourage the use of the best available energy technologies and enforce energy management. For the transport sector, policymakers must encourage the purchase of energy-friendly vehicles. For the building sector, this study suggests that eco-friendly standards (such as low-carbon and energy-saving building materials) be established for home construction. For the power sector, these countries may consider supercritical, ultra-supercritical And Integrated Gasification Combined-Cycle (IGCC) plants, if renewable energy installations are impractical. These policies can form the basis of energy practices and may stimulate a public rebound. 

For the causality results of the Asian new industrial economy, there is a bidirectional causality between economic growth and greenhouse gas emissions and a unidirectional causality running from energy consumption to greenhouse gas emissions. The suggestions for the new Asian industrial economy are similar to those for Southeast Asian countries, including the advice given for each sector above. Individual countries that are part of the new Asian industrial economy have their own energy development policies.

## 5. Conclusions

Worldwide attention to sustainable development has facilitated continuous reductions in ${\mathrm{CO}}_{2}$ emissions in recent decades. Various governments debate issues of economic development and energy consumption especially surrounding fossil fuel use. The key focus of this study was to examine the long-run equilibrium and the existence and direction of causal relationships between greenhouse gas emissions, energy consumption and economic growth in the context of 16 Asian countries for the period 1990–2012. 

The FMOLS method proposed by Pedroni [53] is applied to investigate long-run equilibrium relationships. The FMOLS estimates indicate that there is strong evidence of a long-run relationship between greenhouse gas emissions and energy consumption for the whole sample of 16 Asian countries. A 1% increase in energy consumption increases greenhouse gas emissions: by 0.82% across the whole sample; by 0.18% for the Asian new industrial economies; and by 0.87% for Southeast Asian countries. In addition, a statistically-significant non-linear relationship between greenhouse gas emissions and economic growth is established for the whole sample, as well as for Asian new industrial economies, but an insignificant non-linear relationship between greenhouse gas emissions and economic growth for Southeast Asian countries. 

Finally, we applied a panel error correction model to investigate causal relationships between variables. For the whole sample of 16 countries in Asia, we revealed short-run bidirectional causality between energy consumption and greenhouse gas emissions, between GDP and greenhouse gas emissions and between economic growth and energy consumption. In the Asian newly-industrialized economies, we revealed a short-run unidirectional causality from energy consumption to greenhouse gas emissions and bidirectional causality between greenhouse gas emissions and economic growth; feedback mechanisms are thus implied. For the Southeast Asian nations, we also find the existence of three bidirectional relationships between energy consumption and GDP, between energy consumption and greenhouse gas emissions and between economic growth and greenhouse gas emissions. Those results for Southeast Asian nations suggest that the relevant governments must change their policy approaches in favor of supporting renewable or low-carbon energy use to meet rising energy demands, and adopt more environmentally-benign technology to produce and save energy. With respect to long-run causality, results indicated strong unidirectional causality from economic growth and energy consumption to greenhouse gas emissions for Asian countries and strong unidirectional causality from GDP growth and greenhouse gas emissions to energy consumption for all 16 Asian countries, in the Asian new industrial economy.

With respect to the short-run causality and long-run cointegration relationships in this paper, suggestions for sustainable economic growth in Asian countries revolve around the promotion of energy efficiency and the development of clean renewable energy. 

## Figures and Tables

**Table 1:** Literature on the relationship of the energy-Environment Kuznets Curve (EKC).

Author	Period	Country or Region	Method	Results
Al-Mulali et al. [42]	1981–2011	Vietnam	ARDL model	The EKC hypothesis is not supported
Apergis [19]	1960–2013	15 countries	Quantile cointegration model	The EKC hypothesis is supported for 12 out of 15 countries
Bilgili et al. [22]	1977–2010	17 OECD countries	Panel cointegration, DOLS and FMOLS	The EKC hypothesis is supported
Christopher et al. [20]	1990–2002	47 African countries	OLS estimation	The EKC hypothesis is supported
Duan et al. [37]	1991–2012	China	Generalized least square method	The EKC hypothesis is not supported
Farhani et al. [21]	1990–2010	10 Middle East and North African Countries (MENA)	Panel cointegration, VECM	The EKC hypothesis is supported
Hamit-Haggar [5]	1990–2007	21 Canadian industrial sectors	Panel cointegration test, Granger causality test	The EKC hypothesis is supported
Lean and Smyth [46]	1980–2006	ASEAN	Fisher cointegration, dynamic OLS and VECM Granger causality	The EKC hypothesis is supported
Lee et al. [50]	1980–2001	97 countries	Dynamic GMM method	The EKC hypothesis is supported in America and Europe, but not in Asia and Africa
Li et al. [6]	1996–2012	China	ARDL model, Arellano and Bover (1995) and Blundell and Bond (1998) GMM estimator	The EKC hypothesis is supported
Li and Ma [48]	2003–2011	30 administrative regions in China	OLS estimation	The EKC hypothesis is supported
Olugbenga et al. [25]	1970–2010	8 countries in Asia and Africa	ARDL bounds test, VECM	The EKC hypothesis is supported
Pablo-Romero and Jesús [49]	1990–2011	22 Latin American and Caribbean countries	Panel data analysis	The EKC hypothesis is not supported

Note: 1. FMOLS = ully-Modified ordinary least square LS; ARDL = Autoregressive distributed lag estimation; DOLS = Dynamic ordinary least square; VECM = Vector error correction model; OLS = Ordinary least square; ASEAN = Association of Southeast Asian Nations; OECD = Organization for Economic Co-operation and Development.

**Table 2:** Descriptive statistics.

Variables	Mean	Standard Deviation	Minimum	Maximum
${\mathrm{GHG}}_{i,t}$	1.55	1.03	−1.02	4.05
${\mathrm{GDP}}_{i,t}$	8.38	1.54	5.88	10.80
${\mathrm{ENU}}_{i,t}$	7.08	1.14	4.75	9.18
${\mathrm{GDP}}_{\mathrm{it}}^{2}$	72.66	26.08	34.56	116.63

**Table 3:** Panel unit root test results for all countries.

Variables	IPS Test	CIPS Test
	Statistic	Statistic
${\mathrm{GHG}}_{i,t}$	−1.30	−2.14
${\mathrm{ENU}}_{i,t}$	−1.07	−1.99
${\mathrm{GDP}}_{i,t}$	0.04	−2.08
${\mathrm{GDP}}_{\mathrm{it}}^{2}$	0.40	−1.98
$\Delta {\mathrm{GHG}}_{i,t}$	−5.65 ***	−4.73 ***
$\Delta {\mathrm{ENU}}_{i,t}$	−4.51 ***	−3.84 ***
$\Delta {\mathrm{GDP}}_{i,t}$	−3.73 ***	−2.78 ***
$\Delta {\mathrm{GDP}}_{\mathrm{it}}^{2}$	−3.72 ***	−3.27 ***

Note: “***”, mean that the null hypothesis for the series is rejected at the 1% level. IPS: Im, Pesaran and Shin. CIPS = the cross-sectional augmented panel unit root test.

**Table 4:** Pedroni panel cointegration test results.

Test	Statistic	Test Statistic
Panel statistic	Panel v-statistic	3.89 **
	Panel rho-statistic	−4.39 ***
	Panel pp-statistic	−10.20 ***
	Panel ADF-statistic	−12.46 ***
Group statistic	Group rho-statistic	0.38
	Group pp-statistic	−3.65 ***
	Group ADF-statistic	−6.03 ***

Note: (1) “***”and “**” mean that the null hypothesis for the series is rejected at the 1% and 5% level, respectively. (2) The lag lengths are selected using AIC.

**Table 5:** Fully-modified least squares estimates.

Whole Sample		
Variable	FMOLS	
	**Coefficient**	t−Statistic
${\mathrm{ENU}}_{i,t}$	0.82	6.14 ***
${\mathrm{GDP}}_{i,t}$	0.51	1.36
${\mathrm{GDP}}_{\mathrm{it}}^{2}$	−0.03	1.31
**Asian New Industrial Economy**		
${\mathrm{ENU}}_{i,t}$	0.18	2.48 **
${\mathrm{GDP}}_{i,t}$	8.16	7.34 ***
${\mathrm{GDP}}_{\mathrm{it}}^{2}$	−0.40	−7.31 ***
**Southeast Asian Nations**		
${\mathrm{ENU}}_{i,t}$	0.87	10.76 ***
${\mathrm{GDP}}_{i,t}$	0.22	0.71
${\mathrm{GDP}}_{\mathrm{it}}^{2}$	−0.007	−0.35

Note: 1. “***” and “**” mean that the null hypothesis for the series is rejected at the 1% and 5% level, respectively.

**Table 6:** Panel causality test results.

Region	Source of Causality			
Panel A: All 16 Countries	Short-Run			Long-Run
**Dependent**	ΔGHG	ΔENU	ΔGDP	
ΔGHG	-	$0.29^{\;***}$ (0.00)	$0.37{\;}^{***}$ (0.00)	$-0.08{\;}^{***}$ (0.00)
ΔENU	$0.28^{\;***}$ (0.48)	-	$0.41^{\;***}$ (0.05)	$-0.21{\;}^{***}$ (0.00)
ΔGDP	$0.32{\;}^{**}$ (0.00)	$0.01^{\;*}$ (0.37)	-	$-0.05{\;}^{***}$ (0.35)
**Panel B: Asian New Industrial Economy**				
**Dependent**	ΔGHG	ΔENU	ΔGDP	
ΔGHG	-	$0.41{\;}^{**}$ (0.04)	$0.53^{\;***}$ (0.00)	$-0.18^{\;**}$ (0.02)
ΔENU	−0.01 (0.96)	-	0.13 (0.77)	$-0.34^{\;***}$ (0.00)
ΔGDP	$0.68^{\;**}$ (0.03)	−0.15 (0.49)	-	−0.06 (0.12)
**Panel C: Southeast Asian Nations**				
**Dependent**	ΔGHG	ΔENU	ΔGDP	
ΔGHG	-	$0.75{\;}^{*}$ (0.08)	$0.62{\;}^{*}$ (0.00)	$-0.29{\;}^{***}$ (0.00)
ΔENU	$0.44{\;}^{**}$ (0.04)	-	$0.40^{\;**}$ (0.02)	−0.18 (0.13)
ΔGDP	$0.15^{\;**}$ (0.02)	$0.20^{\;***}$ (0.00)	-	−0.01 (0.63)

Note: “***”, “**” and “*” mean that the null hypothesis for the series is rejected at the 1%, 5% and 10% level, respectively. I added the column heading.
